# Digital technology for health shows disparities in cancer prevention between digital health technology users and the general population in Romania

**DOI:** 10.3389/fonc.2023.1171699

**Published:** 2023-07-06

**Authors:** Bogdan C. Pana, Nicolae Ciufu, Carmen Ciufu, Florentina L. Furtunescu, Adina Turcu-Stiolica, Laura Mazilu

**Affiliations:** ^1^ Department of Public Health, University of Medicine and Pharmacy Carol Davila, Bucharest, Romania; ^2^ Ovidius Clinical Hospital, Constanta, Romania; ^3^ Department of Surgery, Ovidius Clinical Hospital, Constanta, Romania; ^4^ Faculty of Medicine, Ovidius University, Constanta, Romania; ^5^ Department of Imagistic, Ovidius Clinical Hospital, Constanta, Romania; ^6^ Department of Pharmacoeconomics, University of Medicine and Pharmacy, Craiova, Romania; ^7^ Department of Oncology, Ovidius Clinical Hospital, Constanta, Romania

**Keywords:** digital health technologies, prevention, self-care, screening, cancer

## Abstract

**Introduction:**

Digital health services and technology are rapidly developing following the COVID-19 pandemic. This study aimed to reveal the differences between users of digital health technology (DHT) and the general population with regard to cancer prevention.

**Materials and Methods:**

This was an observational study on a conventional sample of 270 DHT users with completed data, performed in September 2021.

**Results:**

A significant difference was observed in the proportion of DHT users and the general population reporting the screening test results, which was 2–6 times higher in the DHT group. Digital technologies applied to the “self-care” model were more suitable for internet-literate populations.

**Discussion:**

Including digital technologies in a self-care model may be more suitable for internet-literate individuals. Thus, in a preventative health organizational framework, DHT should be integrated and used at the primary care level in the general population to improve disparities in the preventative health domain.

## 1 Introduction

Digital healthcare refers to technologies and services that use digital information and communication tools to improve the prevention, diagnosis, treatment, monitoring, and management of health-related issues and monitor and manage lifestyle habits that affect health. Digital health technologies (DHT) for preventive healthcare include wearables, sensors, telemedicine tools, and software applications that can evaluate health risks based on questionnaires and measurements. They can also recommend healthy lifestyle habits, record targeted health measurements (such as weight, glycemia, and cholesterol), and perform screening tests (1, 2).

As the COVID pandemic spread, several individuals chose online health services owing to fear of infection from traditional healthcare settings and convenience as these new technologies have recently become widely available. Consequently, we have witnessed a major rise in the development of digital technologies in the last few years and an increase in healthcare delivery through online settings (3). However, the literature on the topic is scarce, sparse, and immature, and knowledge is building up (4).

However, the rapid pace of development and adoption generates skepticism from the government, policymakers, and health system managers regarding the impact of digital self-care information and services on quality, safety, cost, and inequalities. For example, these services may be able to address some inequalities regarding access to health services. However, they may create other inequalities, such as disparities in the resources or skills needed to take advantage of these new technologies (5–7).

In oncology, applications are included in current recommended therapies (8). Nevertheless, health prevention is essential, and the studies exploring the DHT, particularly telephone reminders or messages, for cancer screening participation found that they do have effect (9, 10). Further research is needed to reveal also the effects on education and other aspects taken into consideration by different stakeholders. In this study, we aimed to build knowledge regarding DHT use for cancer prevention with a view on possible disparities.

The study’s objective was to compare the user profiles and the proportion of cancer preventive measures reported by DHT users using data from the general population in Romania.

## 2 Materials and methods

This was an observational descriptive study on a conventional sample of users of a DHT application, Alprevia© (Panmedica, Romania), over one month (September) in 2021, defined as the “DHT sample.” The application estimates risk, collects previous screening data, and recommends examinations, tests, consultations, and lifestyle changes for cardiovascular diseases, cancer, diabetes, and depression. The application collects no personal identification data.

The indicator used for comparison was: *Proportion of women/men (aged “x -y”) reporting the test “z” in the past years*: “z” is the recommended test (such as mammography and colonoscopy), and “x-y” is the age interval for that test according to the disease-specific guidelines.

The target age groups for specific screening and appropriate screening tests were considered based on European cancer screening/detection guides, as follows:

### 2.1 Women

a) Cervical cancer:
- Pap screening: 20–69 years, (11)
- HPV-DNA screening: 30–69 years, (11)
b) Breast cancer:
- Mammogram: 50–69 years, (10)
- Clinical examination of the breast: 50–69 years, (12)
- Breast ultrasound: 50–69 years, (10)
- Breast self-examination education: 50–69 years, (12)
c) Colorectal cancer
- Colonoscopy: 50–74 years, (11)
- Fecal occult blood test (FOBT): 50–74 years, (13)
- Fecal immunochemical test (FIT): 50–74 years (13).

### 2.2 Men

a) Prostate cancer:
- Prostate-specific antigen (PSA) screening: 55–69 years (14),
- Clinical examination of the prostate: 55–69 years (14),
b) Colorectal cancer
- Colonoscopy: 50–74 years (13),
- FOBT: 50–74 years (13),
- FIT: 50–74 years (13).

The application (for September) recorded the data of 270 participants (men and women) who had assessed their cancer risk. Access was free, with no username, password, or registration required. The general population was invited to use the application through web and Facebook advertisements. No specific groups were invited. We analyzed 261 applications with complete data and calculated the percentage of individuals within the target group that used each screening method.

Descriptive analysis was performed using GraphPad Prism 9.4.1. (GraphPad Software, San Diego, CA, USA). Chi-square or Fisher’s exact two-sided test was used in contingency analysis, with statistical significance defined as p <0.05.

## 3 Results

The DHT conventional sample had the following sex distribution: 28% men and 72% women. Approximately 97% of the group were aged 20–69, and 70% were 35–54 years old. The average age was 43, with a Gaussian distribution of 19–71 years.

The DHT conventional sample differed from the general population, with statistically significant differences in the 25–29 and 65–74 age groups, as presented in **Table 1**.

**Table 1:** Age distribution categories and statistical differences in the general population and DHT sample.

Age category	General population N=13,641,072	DHT sample N=261	p-value
20–24	1,003,110 (7.35%)	13 (4.98%)	0.6803
25–29	1,007,238 (7.38%)	18 (6.90%)	0.0440
30–34	1,337,850 (9.81%)	34 (13.03%)	0.6998
35–39	1,296,368 (9.50%)	30 (11.49%)	0.6198
40–44	1,518,585 (11.13%)	48 (18.39%)	0.1307
45–49	1,478,122 (10.84%)	36 (13.79%)	0.3940
50–54	1,577,308 (11.56%)	40 (15.33%)	0.5122
55–59	1,016,629 (7.45%)	22 (8.43%)	0.6378
60–64	1,230,289 (9.02%)	11 (4.21%)	0.5260
65–69	1,214,300 (8.90%)	8 (3.07%)	0.0295
70–74	961,273 (7.05%)	1 (0.38%)	0.0225

DHT, digital health technologies.

**Figure 1:**
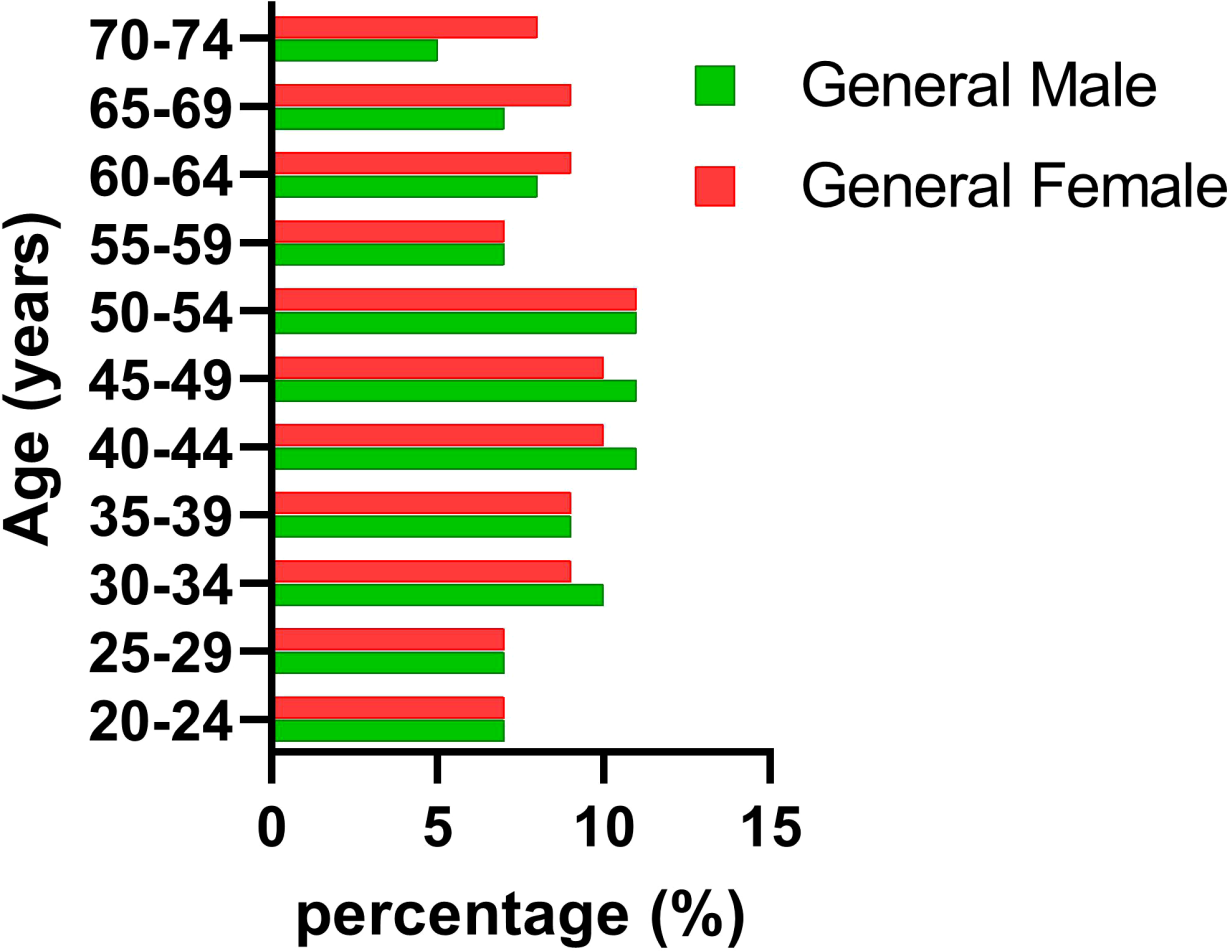
General population age and sex distribution.

**Figure 2:**
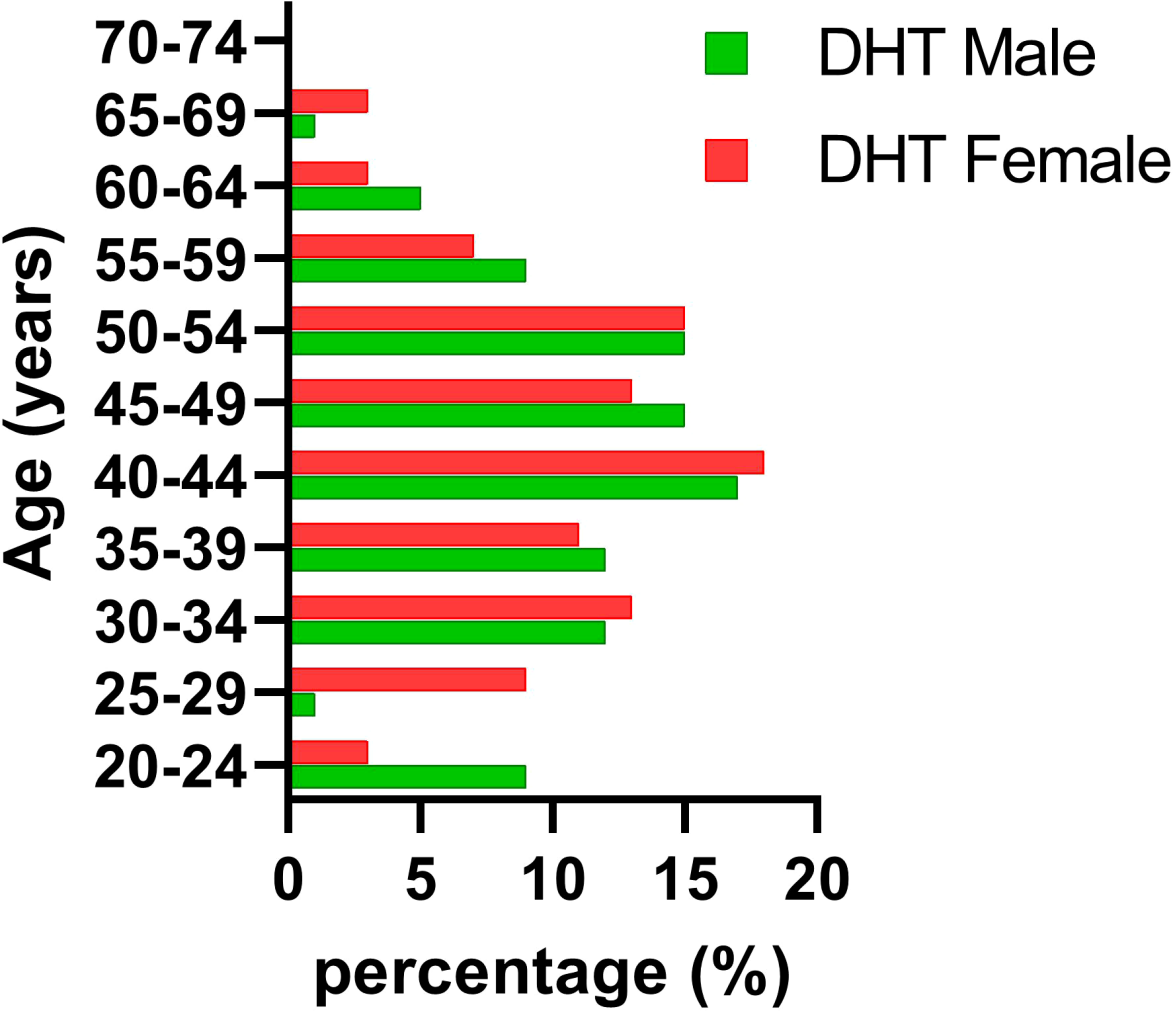
DHT sample age and sex distribution. DHT, digital health technologies.

**Figures 1**, **2** reveal notable differences between men and women; however, these differences were not statistically significant (p = 0.6563 and p = 0.6914, respectively). Our findings also indicated that the DHT group comprised mostly women (72%) aged 30–54 years, which differed from the demographics of the general population, where we observed almost the same percentage of women as men (51%).

We analyzed the proportions of the preventive screening tests recommended for the DHT users (men and women) in each target age group. We compared these with the European Core Health Indicators (ECHI) (15) and European Health Interview Survey (EHIS) (16) data, where available. These comparisons are presented in **Tables 1**–**3**. The numbers of the target group are displayed in the target group column for each screening test.

**Table 2:** Prevalence of recommended preventive tests in women.

Type of cancer	Screening test/history Women	Target age group	N	Proportion within the target group	Romania -ECHI/EHIS-	Europe 27 -Average-
Cervical	Pap screening:	20–69	187	48.13%	27%–11.5%: Low education level 30.2%: Intermediate education level 42.1%: Higher education level	71.8%
	HPV-DNA screening:	30–69	164	17.07%	8.1%	–
Breast:	Mammogram:	50–69	58	29.31%	2.3%	52.4%
	Clinical examination of the breast:	50–69	58	20.69%	6.6%–1.9%: Low education level, 8.9%: Intermediate education level 14.7%: Higher education level	70.1%
	Breast ultrasound:	50–69	58	22.41%	NA*	NA*
	Breast self-examination education	50–69	58	37.93%	NA*	NA*
Colorectal:	Colonoscopy:	50–74	59	13.56%	2.7%	10.4
	Fecal occult blood test	50–74	59	1.69%	4.8%	33.6%
	Fecal immunochemical test	50–74	59	1.69%	NA*	NA*
					3.8%	30.6%

* NA, Not available; ECHI, European Core Health Indicators; EHIS, European Health Interview Survey.

**Table 3:** Prevalence of recommended preventive tests in men.

Type of cancer	Screening test/history Men	Target age group	N	Proportion within the target group	Romania -ECHI-	Europe 27 -Average-
Prostate:	Prostate-specific antigen screening	55–69	12	50.00%	NA*	NA*
	Clinical examination of the prostate	55–69	12	16.67%	NA*	NA*
Colorectal:	Colonoscopy	50–74	23	21.74%	2.1%	10.2%
	Fecal occult blood test	50–74	23	4.35%	3.6%	33.1%
	Fecal immunochemical test	50–74	23	0%	NA*	NA*
					3.5%	30.2%

* NA, Not available; ECHI, European Core Health Indicators; EHIS, European Health Interview Survey.

### 3.1 Women

The cervical cancer tests involved Pap smears every three years and HPV-DNA testing every five years. The proportion of women (20–69 years) who reported having had a Pap test was 48% in DHT users, compared to 27% in Romania and 71.8% in European Union average (includes 27 member states; EU 27) as reported by the ECHI. According to the most recent EHIS performed in Romania in 2019, 37.5% of women reported having had Pap smear screening in the preceding two years, compared to 70.8% in the EU 27.

The proportion of women in our DHT group who reported having been tested for HPV-DNA was 17%, which was much lower than that for Pap, at 48,13%, reflecting the general trend in Romania with regard to HPV testing, at 8.1% (17).

The primary breast cancer standard screening test investigated was mammography; It was recommended at 50 years for women with no family history of breast cancer to be performed every two years until age 69 (12). The proportion of women (aged 50–69 years) who reported having had a mammogram within the preceding two years in the DHT group was 29.3%, compared to 2.3% in Romania and 52.4% in EU 27 (16). The values for other tests recommended or used for breast cancer screening were lower than those in EU 27 (20.6% reported having clinical examinations versus the EU 27 average of 70%). In the target group, 22% of the women underwent breast echography, which has some specific indications but is not considered a general screening method.

Of the women in the DHT group, 13.56% reported having undergone colorectal cancer tests (sigmoidoscopy or colonoscopy). Only 1.69% had undergone FOBTs, and 1.69% had undergone FITs. This was far below the EU 27 average of 30.6% but was four times higher than the percentage reported for the general Romanian population.

### 3.2 Men

PSA testing is a general recommendation for prostate cancer screening, alongside the clinical examination of the prostate. Of the DHT group men, 50% had undergone PSA screening, and 16.7% had undergone clinical examination of the prostate. The data for comparing prostate screening results with those in the EHIS and ECHI databases were unavailable (15, 16).

For colorectal cancer in the DHT group, 21.7% of the men reported having had sigmoidoscopy or colonoscopy, 4.3% had undergone FOBT, and 1.69% had undergone FITs. These figures were comparable to the EU 27 average of 30.2%. However, they were six times higher than those of the general population in Romania.

## 4 Discussion

In Romania, 80% of the population has internet access (18) from a highly developed and competitive infrastructure. Romania ranks tenth out of 180 countries in the average fixed broadband speed, with a speed of 174.26 Mbps reported in April 2023, which is twice the global 80.12 Mbps average. On mobile device the average upload speed it was 49.03 Mbps average, ranking 44 out of 138 global positions list, above global performance of 42.07 Mbps (19) One in every two Romanians owns a smartphone (20). Therefore, devices and internet connections are not barriers to accessing DHT in Romania.

For all cancer screening tests we investigated, the proportion of users who reported having undergone screening tests was two to six times higher in the DHT group than in the general Romanian population but closely mirrored the European average. Additionally, the age distribution reveals a large proportion of users <40 years, indicating that DHT can help detect cancers early in these age groups in the future, which can be considered a strength of DHT in oncology.

Challenges and barriers exist in balancing digital self-care utilization and general accessibility. Primary care centers are usually the first point of contact in prevention, and whether digital health will change this arrangement is questionable (21).

Herein, we revealed that it is not technical barriers but rather educational ones that influence the use of DHT for cancer prevention.

## 5 Conclusions

A significant difference existed between DHT users and the general Romanian population in terms of accessing cancer prevention screening tests. The DHT users in our cohort were mostly women (72%) aged 30–54 years. Regarding the utilization of preventive cancer screening tests, the DHT group reported two to six times higher rates compared to the general Romanian population. This led us to conclude that digital technologies in the “self-care” model were more amenable for internet-literate individuals with higher levels of education. This group also had the highest rate of utilizing screening tests in the general European population, according to the ECHI and EHIS. Consequently, implications in terms of the healthcare prevention organizational framework are that DHT should be integrated and used at the primary care level to have a stronger impact on the general population—older and younger—with different levels of education and internet literacy and to address health disparities in the preventive health domain.

## Data availability statement

The datasets presented in this study can be found in online repositories. The names of the repository/repositories and accession number(s) can be found below: https://osf.io/tjuqe/files/osfstorage/63e1edd93db09501351ced85.

## Ethics statement

The studies involving human participants were reviewed and approved by Ovidius Clinical Hospital Medical Ethics and Research Comity with Decision No. 5.1./15.08.2021. Written informed consent from the participants was not required to participate in this study in accordance with the national legislation and the institutional requirements.

## Author contributions

BP, NC, and CC contributed to the study’s conception, methodology, original draft preparation, and design. These authors contributed equally to this work and share the first authorship. AT-S performed the statistical analysis. FF and LM reviewed the draft of the manuscript. All authors contributed to the manuscript revision and read and approved the final version.
